# Psychometric Evaluation of the Workstyle Short Form among Nursing Assistants with Work-Related Musculoskeletal Symptoms

**DOI:** 10.3390/ijerph15040823

**Published:** 2018-04-22

**Authors:** Kin Cheung, Shirley S. Y. Ching, Ka Yan Ma, Grace Szeto

**Affiliations:** 1 School of Nursing, The Hong Kong Polytechnic University, Hong Kong, China; shirley.ching@polyu.edu.hk (S.S.Y.C.); eunis.ma@polyu.edu.hk (K.Y.M.); 2 School of Medical and Health Sciences, The Tung Wah College, Hong Kong, China; graceszeto@twc.edu.hk

**Keywords:** workstyle, factor analysis, non-sedentary workforce, work-related musculoskeletal symptoms, construct validity

## Abstract

The Workstyle Short Form (24 items) (WSF-24) has been tested for its psychometric properties on work-related upper-extremity musculoskeletal symptoms (WRUEMSs) among office workers. However, the impact of workstyle should not only be limited to WRUEMSs and the sedentary workforce. The purpose of this study was to test the psychometric properties of the modified 24-item Chinese WSF (C-WSF-24) to identify work-related musculoskeletal symptoms (WRMSs) in various body parts among nursing assistants (NAs) working in nursing homes. Four hundred and thirty-nine NAs participated in the study. The results of the factor analysis were that a four-factor solution (working through pain, social reactivity at work, demands at work and breaks) accounted for 56.45% of the total variance. Furthermore, validation against known groups showed that the total score and subscale scores of the C-WSF-24 had the ability to discriminate between NAs with and without WRMSs in various body parts (such as low back and lower extremities). Additionally, C-WSF-24 had a statistically significant association with the contributing factors to WRMSs. This is the first study to examine the psychometric properties of the C-WSF-24 in the non-sedentary workforce, with a focus on various body parts of WRMSs. The results demonstrated that C-WSF-24 is reliable and valid for assessing WRMSs in various body parts among NAs.

## 1. Introduction

Globally, work-related musculoskeletal symptoms (WRMSs) are a major public health issue [1]. This issue has been shown as one of the leading reasons for nursing personnel to quit their job [2,3]. In nursing homes, this issue not only affects workers’ health (particularly nursing assistants (NAs), direct care providers), but also their clients’ quality of care [2]. Factors contributing to WRMSs have been well studied and include personal, physical, ergonomic, organizational, and psychosocial (involving workstyles) issues [4,5,6,7]. A workstyle is how a worker responds to hectic work demands behaviorally, cognitively and physiologically [5]. According to the workstyle model [8], the behavior component is a response to prolonged forceful movements, awkward postures, or taking limited work breaks. The cognitive component is responsible for increased fear of losing a job, or fear of poor performance. The physiological component is concerned with increased levels of fatigue, pain or functional limitations of various body parts [8,9]. Excessive work demands raise levels of ergonomic and psychological risk factors and trigger adverse workstyles [5]. Workers with adverse workstyles may have tendencies to continue working despite pain, work with insufficient rest breaks, have poor interpersonal relationships with colleagues, or perceive themselves not able to give feedback to supervisors about their heavy workloads [10]. As a result, adverse workstyles can lead to the development of WRMSs, and functional limitations in workers [11]. Consequently, WRMSs among NAs in nursing homes would have negative impacts on their residents’ quality of care. 

Because of the serious impacts of WRMSs, the past decade has seen studies in workstyles and WRMSs. The main focus has been on upper extremities of office workers in the United States (US) [8,11], The Netherlands [12], India [13], Malaysia [10,14,15], Australia [15], and Chinese cooks in Hong Kong (HK) [16]. These studies have adopted the Workstyle Short Form (WSF) (24 or 32 items) [5,6,8,11]. The WSF (24 items) has been translated from English to Chinese [16] and Malay [10]. Only two studies have extended the modified WSF (11 items) to other body parts, such as surgeons’ upper and lower backs [17], and community registered nurses’ lower backs and knees [18] in HK. However, the 24-item WSF has not been extended to other body parts in healthcare workers. NAs working in nursing homes have been identified as a vulnerable workforce for WRMSs [19,20,21]. A validated questionnaire is needed to examine their workstyles so that appropriate strategies can be implemented to reduce their WRMSs. Thus, this study aimed to test the psychometric properties of the modified 24-item Chinese WSF (C-WSF-24) for use with various body parts of NAs working in nursing homes.

## 2. Methods

### 2.1. Study Design and Participants

NAs working in nursing homes were recruited to cover all three regions of HK using convenience sampling. Full-time NAs providing direct resident care for at least one year were invited using different methods in 2014–2015. For instance, the officers in charge of nursing homes were approached by fax, phone, email or face-to-face meetings. With the permission from the officers in charge, NAs were invited to complete the questionnaire within an hour during their working hours. A total of 47 nursing homes with 522 NAs completed the questionnaires. Eighty-two questionnaires were invalid and excluded. The response rate from each participating nursing home was more than 60%. Detailed explanations of the study method have been reported elsewhere [2]. The study was approved by the Institutional Review Board of the HK Polytechnic University. 

### 2.2. Measurements

The study questionnaire included scales which have been tested for their reliability and validity, internationally and locally [22,23,24]. The content validity index of the questionnaire, evaluated by a panel of four experts in regard to item relevancy, was 0.99 [2]. 

#### 2.2.1. Workstyle

This study adopted the C-WSF-24 with five subscales: working through pain (6 items), social reactivity (5 items), workplace stressors (8 items), self-imposed workpace/workload (3 items) and breaks (2 items), measured by a five-point Likert scale (0 = almost never while 4 = almost always) [16]. For the purpose of this psychometric test, these four items specifying the affected body parts were modified to involve all body parts instead of only upper extremities. For instance, the original item of “There really isn’t much I can do to help myself in terms of eliminating or reducing my symptoms in my hands/arms/shoulders/neck” [16] was modified to “… in my neck/shoulders/hands/arms/back/hips/thighs/calves/feet”. The total score and sums of these modified C-WSF-24 subscales were used for the data analyses [2]. 

#### 2.2.2. WRMSs and Severity Level

The Standardized Nordic Musculoskeletal Questionnaire (NMQ) was adopted to assess if the NAs had work-related pain, aches or discomfort in various body parts at the time of the survey [25]. Likewise, the severity of each body part’s symptoms in the previous month were measured by a 5-point Likert scale (1 = very light while 5 = very serious). The sum of the severity level of all body-part symptoms = was calculated, with high scores denoting greater seriousness of the overall WRMSs [2]. 

#### 2.2.3. Functional Limitations and Bothersome Level

The 14-item Short Musculoskeletal Functional Assessment Questionnaire (SMFAQ) [26] was used to assess how much the NAs were bothered by the WRMSs in their daily activities using a 5-point Likert scale (0 = not at all bothered and 4 = extremely bothered). For instance, they were asked “How much are you bothered by WRMSs while bathing, dressing, toileting, or other personal care?” Convergent and group different construct validity have been established for this instrument, with Cronbach’s alpha scores (measuring internal consistency of the scale reliability [27]) of 0.92 and 0.95 respectively [26]. In the present study, the Cronbach’s alpha score was 0.94. The sum of the scale was calculated, with high scores indicating more functional limitations. As well, one item was used to assess how bothersome the WRMSs were to the NAs using a 5-point Likert scale (0 = not at all bothered and 5 = extremely bothered).

#### 2.2.4. Perceived Frequency and Physical Exertion (PE) of Job Tasks

Twenty-two frequently performed job tasks were identified from the literature [28,29,30]. These job tasks included transferring, repositioning, dressing or undressing, bathing, lifting and carrying meal trays, and others. The NAs were first asked the frequency of performing those job tasks using a four-point Likert scale (1 = never and 4 = always). Then, they were asked to rate the PEs of performing each task using a scale from zero (nothing at all) to ten (extremely strong) [31]. In the present study, the Cronbach’s alpha for frequency was 0.87 while that for the PE was 0.95. The sum of the 22-job task measure and the sum of the PE scale were calculated [24], with high scores on each measure representing high frequencies, and greater exertion in performing job tasks, respectively. 

#### 2.2.5. Perceived Ergonomic Exposures (EEs) 

Three subscales (i.e., contribution to WRMSs; frequency of encounters and posture frequency) were adopted to measure the perceived EEs. Based on our previous study [24], nine items were used to evaluate (1) if those EEs contributed to their WRMSs (with dichotomous responses: yes or no); and (2) NAs’ perceived frequencies of encountering these EEs (measured by a four-point Likert scale from 1 = never to 4 = always). These nine items addressed commonly identified EEs such as repetitive movements. Additionally, 10 items were used to ask the NAs about their frequencies of performing different postures such as bending forward, twisting the body more than 45 degrees, and others; these items were measured by a four-point Likert scale (1 = never and 4 = always). In the current study, the Cronbach’s alpha score was 0.82 for EE’s contribution to WRMSs; 0.83 for the frequency of EE encounters; and 0.75 for the frequency of EE postures. The sum of each subscale was calculated, with high scores indicating that the EEs were related to their WRMSs, a high frequency of encountering EEs, and a high posture-encountering frequency, respectively.

#### 2.2.6. Job Content Questionnaire (JCQ)

Seven subscales of JCQ [32] were adopted to measure the psychological job demand (9 items), physical job demand (5 items), supervisor support (4 items), co-worker support (5 items), residents and family support (4 items), decision authority (3 items) and skill discretion (6 items). A four-point Likert scale ranging from 0 = strongly disagree to 4 = strongly agree was used [33]. The Cronbach’s alpha score was 0.75. The sum of each subscale was calculated, with high scores denoting high values of the measures.

#### 2.2.7. Other Factors Associated with WRMSs

Job stress and satisfaction were measured by one individual item—the NAs were asked if they were satisfied or felt stressed with their work, using a 4-point Likert scale (0 = very unsatisfied/not at all stressful and 4 = very satisfied/very stressful). Intention to leave was assessed with dichotomous responses to whether the NAs had thought of quitting the job. Additionally, the NAs were asked to assess their current work ability from zero (completely no work ability) to ten (completely full ability) [2].

### 2.3. Data Analyses

IBM SPSS statistics version 23 was used to analyze the data. Besides descriptive statistics, the construct validity of the modified C-WSF-24 was assessed by three methods: (1) exploratory factor analysis using the principal component extraction method, with an eigenvalue >1 to retain the factor (which provides more variance among measured variables within a factor than eigenvalue ≤1 [34]), followed by varimax rotation to explore its factor structure; (2) known-groups validity [27] to test the hypothesis—the mean scores of the total score or subscales of the modified C-WSF-24 for NAs with WRMSs would be higher than those without WRMSs; and (3) evaluating the relationship between the modified C-WSF-24, and factors contributing to WRMSs and impacts of WRMSs. Testing for normality using the Shapiro–Wilk test found that the modified C-WSF-24 scores were not normally distributed. Non-parametric statistics such as Spearman’s rho and the Mann–Whitney U test were used, with the level of significance set at *p* < 0.05. 

All subjects gave their informed consent for inclusion before they participated in the study. The study was conducted in accordance with the Declaration of Helsinki, and the protocol was approved by the Ethics Committee of the HK Polytechnic University (Project identification code HSEARS20130301003-05).

## 3. Results

### 3.1. Participants’ Characteristics 

Table 1 shows the participants’ characteristics. 439 NAs from 47 nursing homes completed the modified C-WSF-24 items [2]. The majority of them were female, married, and with secondary school education. They had an average working experience of 10.4 years and they represented the aging workforce, with an average age of 51.1 years. Although more than 95% of them were satisfied with their jobs, about 70% of them found their jobs stressful and about 35% had thought of leaving their current job. 

### 3.2. Construct Validity: Factor Analysis

Table 2 shows the results of the factor analysis. The significant value (*p* < 0.001) for Bartlett’s test of sphericity showed that there were some relationships between the items; thus, factor analysis was appropriate for the collected data. In addition, the Kaiser–Meyer–Olkin test score showing a sampling adequacy of 0.92 indicated a high probability of yielding distinct and reliable factors [35]. A four-factor solution accounted for 56.45% of the total variance, with eigenvalues greater than one. Four factors were named, based on the main themes of the loaded items and drawn from the relevant literature [10,11,16].

### 3.3. Construct Validity: Known-Groups Validity

Table 3 shows that the total score and subscale scores of the modified C-WSF-24 had the ability to discriminate between NAs with and without WRMSs in various body parts, except for the upper back. Additionally, NAs with WRMSs experienced higher levels of adverse workstyles than those without WRMSs in various body parts, except for the feet (NAs with feet WRMSs took less breaks than their counterparts). 

### 3.4. Construct Validity: Relationships between the Modified C-WSF-24 and WRMSs’ Contributing Factors

Table 4 and Table 5 illustrate the relationship between the total score of the modified C-WSF-24 and its subscales to the existing measures of WRMS contributing factors. In general, the scores of the modified C-WSF-24 were associated positively with (1) the severity levels, bother levels and functional limitations of the WRMS; (2) the job task frequency, physical exertion involved in job tasks; ergonomic exposure, job stress, intention to leave, and JCQ physical and psychological job demands. Furthermore, the scores were related inversely to job satisfaction and the expectation to work in the subsequent two years. Interestingly, the subscale “Breaks” was not correlated with any of the contributing factors.

## 4. Discussion

To our knowledge, this was the first study to evaluate the psychometric properties of WSF in NAs working in nursing homes as well as involving all body parts of WRMSs. WSF has been well tested, with most studies having focused on office workers’ upper extremity WRMSs in sedentary workers [8,10,11,16]. Our study results demonstrate that the modified C-WSF-24 is also applicable to healthcare workers and various body parts of WRMSs. The modified C-WSF-24 is interdependent and homogenous in terms of the constructs it measures, as illustrated by the high Cronbach’s α, ranging from 0.86 to 0.93 (except for “Breaks” with Cronbach’s α of 0.55). For “Working through pain”, the Cronbach’s α in our study involving the upper and lower extremities and back was 0.87, which is comparable with the original English, HK (α = 0.84) and Malaysian (α = 0.84) WSFs [8,10,16]. Similar findings applied to “Social reactivity at work” with a Cronbach’s α of 0.93, which was higher than the values shown in HK (0.91) [16] and Malaysian (0.81) studies [10]. Additionally, “Demands at work” was loaded in our study as a factor similar to that in the Malaysian study [10] with a comparable Cronbach’s α. 

However, the findings for the factor “Breaks” were inconsistent with other studies. The internal consistency of 0.55 for “Breaks” in our study was the lowest of the subscales and lower than in other studies [8,10,11,16], although it was comparable to another HK study of Chinese cooks (α = 0.65) [16]. The results from the HK workers seem lower than those from computer workers in the Malaysian study (Cronbach’s α = 0.83). These inconsistent results could be partially due to the subscale containing only two items. This is because the magnitude of the inter-item correlations and the number of items in a scale both affect the results of the Cronbach’s α [35,36]. Furthermore, the Chinese working culture in nursing homes might be another reason. NAs might have different views about “Breaks”. In our qualitative study, some NAs stated they were on call during their breaks while some did not have scheduled breaks [28]. Nevertheless, the corrected item total correlation for “Breaks” in our study was 0.38, which was greater than 0.3, indicating that each of these two items correlated well with the total of this subscale [35]. Another interesting finding was the positive loading of “Breaks” found in our study, which was consistent with the Malaysian office workers study [10], but different from office workers in the US [8,11] and the HK Chinese cooks study [16]. This positive loading suggests that these two items are positively associated with an “adverse workstyle” [8,16]. Future studies can be conducted to explore the concept of taking a break at work among different kinds of workers, different work cultures, and in different countries. Cross-cultural comparisons could provide more useful information about the impact of workstyle and its associations with WRMSs in various countries worldwide. 

Regarding the factorial structure, the factors found in our modified C-WSF-24 were similar to those of the original English [8,11], Chinese [16] and Malaysian [10] WSFs in most aspects. The first factor consisted of six items measuring “Working through pain”, which was consistent with both the original English WSF and the C-WSF. For the second factor, “Social reactivity at work”, the item 11 (“If I take a break from work for relaxation or physical exercise, my colleagues/boss will be unhappy with me”) was not loaded onto this factor in our study, which was different from both the original English WSF and the C-WSF. Instead, it was loaded on the third factor “Demands at work”. Item 11 was loaded to either “Social reactivity at work” in the original English and Chinese studies or “Working through pain” in the Malaysian study. Twenty-two items (items 11–22) were grouped together to form the third factor, which was similar to the Malaysian study (items 12–22). This third factor was distributed into three different factors: “Limited workplace support”, “Workplace stressors” and “Self-imposed workpace/workload” in the original English WSF and distributed into two factors “Deadlines/pressure” and “Self-imposed workpace/workload” in C-WSF. The fourth factor “Breaks” captured the two items which were consistent with all the other studies. Although the factors identified in the four studies were comparable, it is suggested that more studies are needed to examine the constructs of workstyle further. These constructs might be affected by the working culture, type of work, equipment used, physical and psychological work demands, country of work and ethnic groups [16]. For instance, the ergonomic hazards faced by NAs working in nursing homes would be different from cooks and office workers. NAs cannot handle residents like objects by packing them in a way for easy moving; rather, NAs need to seek cooperation from residents [37]. 

We further demonstrated the good construct validity of the modified C-WSF-24 by showing that most WRMS contributing factors were correlated with the total score and subscales. These results further confirmed Feuerstein’s workstyle conceptual framework [5,6]. For NAs working in nursing homes, their adverse workstyles might be associated with the WRMSs’ contributing factors, such as frequently performing job tasks with increased physical exertion, and frequent exposure to ergonomic stressors, and physical and psychological job demands. Our qualitative study results further supported these assumptions—NAs had to continue to work even they had WRMSs [28]. As a result, with the increased physical and psychological stressors, the frequency, intensity and duration of the adverse workstyles increased as well. This vicious cycle may exacerbate the development of WRMSs in different body parts. Thus, our study found that NAs with more adverse workstyles were more likely to report WRMSs with an increased level of severity, functional limitations, job dissatisfaction, intention to leave the job, and perceived inability to work in the next two years. This first study provides a foundation and promising results for applying the modified C-WSF-24 to healthcare workers and various body parts of WRMSs. The inability to discriminate between NAs with and without WRMSs in the upper back might be due to the small number of NAs (*n* = 28, 6.4%) [2] with upper back WRMSs. More studies are needed to further test the instrument’s psychometric properties across different work populations in various countries. A validated, modified C-WSF-24 is essential to allow the assessment of adverse workstyles to further prevent WRMSs.

The findings of our study should be interpreted with caution because of several limitations. Convenience sampling was employed, which could contribute to selection bias. Since the samples came from three regions of HK and covered similar percentages of non-governmental organizations and private nursing homes [2], it is likely that the study sample was representative. In addition, the WRMSs and their associated factors were cross-sectional data which cannot suggest causal relationships. However, factor analysis and construct validity testing of instruments are normally performed using cross-sectional data for the purpose of determining psychometric properties, instead of causal relationships. Furthermore, the self-reported data were subjected to response and recall biases. 

## 5. Conclusions

This is the first study to examine the psychometric properties of the modified C-WSF-24 in NAs working in nursing homes, with a focus on various body parts of WRMSs. The results are encouraging, with acceptable internal consistency and good construct validity. The modified C-WSF-24 can be used to assess adverse workstyles of healthcare workers and the effects of WRMSs on various body parts of WRMSs. Future studies are recommended to explore the concept of taking breaks, to consolidate the constructs of workstyles and to identify maladaptive workstyles which can further prevent WRMSs in various body parts across different working populations in various countries. 

## Figures and Tables

**Table 1:** Personal and work characteristics of nursing assistants (NAs) (*N* = 439).

Characteristics	Number (%)
Gender	*N* = 439
Female	430 (97.9%)
Male	9 (2.1%)
Marital Status	*N* = 439
Single	36 (8.2%)
Married	350 (79.7%)
Divorced	22 (5.0%)
Widow	29 (6.6%)
Others	2 (0.5%)
Education Level	*N* = 433
Primary School	142 (32.8%)
Secondary School	276 (63.7%)
Post-Secondary	13 (3.0%)
University	2 (0.5%)
Self-Rated Health	*N* = 439
Very Good	2 (0.5%)
Good	57 (13.0%)
Fair	174 (39.6%)
Not Good	197 (44.9%)
Very Bad	9 (2.1%)
Job Title	*N* = 439
Personal Care Workers	399 (90.9%)
Health Workers	40 (9.1%)
Work Overtime	*N* = 438
Yes	111 (25.3%)
No	327 (74.7%)
Job Satisfaction	*N* = 437
Very Satisfactory	71 (16.2%)
Satisfactory	347 (79.4%)
Not Satisfactory	19 (4.3%)
Very Unsatisfactory	0 (0.0%)
Job Stress	*N* = 438
Very Stressful	20 (4.6%)
Stressful	284 (64.8%)
Not Stressful	125 (28.5%)
Not Very Stressful	9 (2.1%)
Intention to Leave	*N* = 437
Yes	154 (35.2%)
No	283 (64.8%)
Work in the Next 2 Years	*N* = 429
Very Sure	318 (74.1%)
Not Sure	107 (24.9%)
Impossible	4 (0.9%)
	Mean ± SD (Range)
	*N* = 438
	51.1 ± 9.6 (19–69)
Working Experience (years)	*N* = 439
	10.4 ± 6.4 (1–36)
Work Ability (0–10)	*N* = 438
	7.8 ± 1.5 (2–10)

Percentages may not add up to 100% due to rounding.

**Table 2:** Factor structure of the modified C-WSF-24 (workstyle short form) workstyle measure after principal component analysis using varimax rotation.

	Questions	Factor Loading	Variance	Cronbach’s α
Working through pain (#1–6)			35.06	0.87
1.	I keep working when I feel aching or discomfort, so that the quality of my work will not be affected. 我會在疼痛、不舒適的情況下繼續工作，這樣才不會影響我的工作質量。	0.73		
2.	My neck/shoulders/hands/arms/back/hips/thighs/calves/feet get tired at work. 在工作時，我的頸/肩膊/雙手/雙臂/背/臀/大小腿/膝蓋/脚會感到疲勞。	0.76		
3.	I feel aching while at work 在工作時，我會感到疼痛。	0.80		
4.	As I do not know how to relieve my aching neck/shoulders/hands/arms/back/hips/ thighs/calves/feet, I keep on working with pain. 因為我對自己的頸/上肢/背/下肢痛毫無辦法，所以只能忍痛繼續工作。	0.83		
5.	I do not know how to eliminate or relieve various symptoms of my neck/shoulders/hands/arms/back/hips/thighs/calves/feet. 我實在沒有辦法消除或緩解自己頸/上肢/背/下肢所出現的各種症狀。	0.71		
6.	My neck/upper extremities/back/lower extremities (over or more places) may make some abrupt, fierce, fast or sudden movements. 我的頸/上肢/背/下肢（其中一處或多處）會做一些急促、猛烈、快速、突然的動作。	0.55		
Social reactivity at work (#7–10)			9.00	0.93
7.	I cannot interrupt my work because my other team members will be unhappy with me. 我不能中途停工，因為這樣做會讓其他同事對我有意見。	0.78		
8.	I cannot interrupt my work, because it would disappoint my boss or increase his/her responsibility. 我不能中途停工，因為這樣做會讓上司失望或增加他的負擔。	0.87		
9.	I cannot interrupt my work, because it would disappoint my colleagues or increase their workload. 我不能中途停工，因為這樣做會讓同事失望或增加他們的負擔	0.85		
10.	I cannot interrupt my work, because it would affect my appraisal, promotion, and/or cause me to lose my job. 我不能中途停工，因為這樣做會影響我的評估、晉升，和/或讓我丟掉工作。	0.84		
Demands at work (#11–22)			6.54	0.86
11.	If I take a break from work for relaxation or physical exercises, my colleagues/boss will be unhappy with me. 如果我關心自己的健康而放下工作，放鬆一下或做做運動，我的同事/上司會對我有意見。	0.50		
12.	Although I have worked very hard, I still do not know whether my work has been duly recognized. 儘管我在工作中付出了很大努力，但我還是真的不知道我的工作是否得到應有的認同。	0.58		
13.	If I have not finished my work, my boss will give me a hard time. 如果我沒完成自己的工作，上司不會讓我好過。	0.51		
14.	If I communicate some problems to the supervisor—for instance, that some coworkers have not been working hard enough—this has no effect, so I might as well just work harder. 如果我向主管反映（一些）問題，比如某某同事沒有努力做好自己的本職工作，這根本起不了什麼作用，所以不如自己做多點。	0.57		
15.	I feel very depressed, as my boss’ expectations on the quality of work are different from mine. 上司或其他人對工作質量的要求與我不同，這一點讓我感到很沮喪。	0.72		
16.	I have too many deadlines and I can never finish my work. 我的工作有太多的最後期限，所以工作總是做不完。	0.69		
17.	Although I arrange my work in good order so that I can finish it before the deadline, things change so frequently that I have to work even harder to finish it on time. 儘管我會有條理地安排自己的工作，以便能夠在最後期限前完成工作，但情況在不斷變化，自己還是要更加努力地工作，以便按時完成。	0.52		
18.	My work schedule is hard to control. 我的工作時間表很難控制。	0.67		
19.	I feel pressure at work. 我在工作時會感到壓力。	0.63		
20.	I motivate myself to work harder and set up targets that are higher than those my boss and other colleagues have expected. 工作中我會敦促自己，確立自己的表現高於上司和其他人的預期目標。	0.36		
21.	If my colleagues fail to do their work, I have to assume more responsibilities. 我的同事沒有做好自己的份內工作，我就得承擔更多的工作。	0.60		
22.	Others tell me to slow down and not to work so intensely. 別人會告訴我應該放慢節奏，工作不要那麼拼命。	0.45		
Breaks (#23–24)			5.85	0.55
23.	During my regular workday I take breaks to do some stretches. 在日常工作期間，我會中途停下來休息，做做伸展運動。	0.78		
24.	While at work I occasionally stop working to take a break. 在工作時，我會不時停下來休息。	0.77		

**Table 3:** Four factors identified from the factor analysis of C-WSF-24 items with a total variance of 56.5% and their known-groups validity to discriminate between NAs with WRMSs and without WRMSs in various body parts using the Mann–Whitney U test (*N* = 439).

Name of Factors	Body Parts	With WRMSs	Without WRMSs	Mann–Whitney U	*p*-Value
(Items #)	*N* (with WRMSs, without WRMSs)	Mean (SD)	Mean (SD)		
Working through Pain					
(1–6)	Neck, 439 (108, 331)	10.62 (4.71)	8.65 (5.30)	13,600.5	<0.0001 ***
	Shoulder, 439 (232, 207)	10.35 (4.79)	7.77 (5.37)	16,864.5	<0.0001 ***
	Elbow, 439 (121, 318)	11.62 (5.01)	8.19 (5.00)	12,177.5	<0.0001 ***
	Wrist, 439 (96, 343)	11.34 (4.79)	8.51 (5.18)	11,260.5	<0.0001 ***
	Fingers, 439 (112, 327)	10.82 (5.11)	8.55 (5.14)	13,909.5	<0.0001 ***
	Low back, 439 (182, 257)	10.71 (5.40)	8.01 (4.80)	16,660.5	<0.0001 ***
	Hip/thigh, 439 (51, 388)	11.71 (5.14)	8.79 (5.15)	6605.5	<0.0001 ***
	Knee, 439 (165, 274)	11.01 (5.25)	8.00 (4.88)	15,236.5	<0.0001 ***
	Calf, 439 (78, 361)	10.69 (5.46)	8.80 (5.12)	11,099.5	0.003 **
	Feet, 439 (124, 315)	10.91 (5.29)	8.43 (5.04)	14,393.5	<0.0001 ***
	At least 1, 439 (388, 51)	9.77 (5.06)	4.25 (3.62)	3730	<0.0001 ***
Social reactivity at work					
(7–10)	Elbow, 439 (121, 318)	3.96 4.10)	3.18 (4.17)	16,605	0.022 *
	Hip/thigh, 439 (51, 388)	4.61 (4.62)	3.23 (4.07)	7923	0.017 *
	Knee, 439 (165, 274)	3.96 (4.41)	3.05 (3.97)	19,574	0.015 *
	Feet, 439 (124, 315)	4.13 (4.45)	3.10 (4.01)	16,574.5	0.011 *
	At least 1, 439 (388, 51)	3.55 (4.25)	2.20 (3.15)	8033	0.024 *
Demands at work					
(11–22)	Elbow, 439 (121, 318)	14.17 (9.23)	11.87 (8.33)	16,433	0.018 *
	Wrist, 439 (96, 343)	14.34 (9.48)	11.99 (8.34)	14,064	0.029 *
	Fingers, 439 (112, 327)	14.34 (8.91)	11.87 (8.47)	15,218	0.008 **
	Hip/thigh, 439 (51, 388)	16.08 (10.10)	12.03 (8.33)	7400	0.003 **
	Knee, 439 (165, 274)	14.88 (9.50)	11.07 (7.75)	17,148	<0.0001 ***
	At least 1, 399 (388, 51)	12.94 (8.73)	9.16 (7.17)	7243.5	0.002 **
Breaks					
(23–24)	Shoulder, 439 (232, 207)	1.69 (1.63)	1.39 (1.62)	21,091	0.02 *
	Feet, 439 (124, 315)	1.38 (1.75)	1.61 (1.58)	17,176.5	0.041 *
Workstyle Total Score	Neck, 439 (108, 331)	29.69 (14.77)	25.56 (15.56)	14,620	0.004 **
	Shoulder, 439 (232, 207)	28.42 (14.67)	24.51 (16.07)	19,896.5	0.002 **
	Elbow, 439 (121, 318)	31.46 (15.89)	24.71 (14.89)	14,303.5	<0.0001 ***
	Wrist, 439 (96, 343)	31.14 (15.58)	25.30 (15.20)	12,713	0.001 **
	Fingers, 439 (112, 327)	30.27 (14.68)	25.31 (15.53)	14,538.5	0.001 **
	Lower back, 439 (182, 257)	29.63 (16.77)	24.41 (14.09)	19,338.5	0.002 **
	Hip/thigh, 439 (51, 388)	33.90 (17.16)	25.61 (14.98)	6936.5	0.001 **
	Knee, 439 (165, 274)	31.33 (16.84)	23.71 (13.82)	16,639.5	<0.0001 ***
	Calf, 439 (78, 361)	30.44 (16.73)	25.74 (15.06)	11,805.5	0.025 *
	Feet, 439 (124, 315)	29.98 (15.69)	25.23 (15.18)	15,892.5	0.002 **
	At least 1, 439 (388, 51)	27.82 (15.49)	17.10 (11.47)	5719.5	<0.0001 ***

Note: Upper back: 339 (411, 28); does not significantly discriminate between NAs with and without WRMSs in all body parts; * *p* < 0.05; ** *p* < 0.01; *** *p* < 0.001.

**Table 4:** Correlations between C-WSF-24 and WRMSs’ contributing factors by non-parametric Spearman’s rho.

	1	2	3	4	5	6	7	8	9	10	11	12	13	14	15	16	17	18
1. WTP	-	0.5 **	0.52 **	−0.001	0.78 **	0.39 **	0.39 **	0.48 **	0.24 **	0.38 **	0.40 **	0.35 **	0.44 **	−0.20 **	0.35 **	−0.01	0.33 **	−0.20 **
2. SR		-	0.63 **	0.06	0.77 *	0.16 **	0.17 **	0.29 **	0.09	0.27 **	0.30 **	0.18 **	0.26 **	−0.13 **	0.23 **	−0.004	−0.27 **	−0.15 **
3. DW			-	0.11 *	0.90 **	0.18 **	0.26 **	0.26 **	0.18 **	0.34 **	0.32 **	0.27 **	0.33 **	−0.20 **	0.34 **	−0.04	0.27 **	−0.15 **
4. Breaks				-	0.17 **	−0.06	−0.06	−0.04	0.06	0.04	0.01	−0.01	0.03	0.10 **	−0.03	−0.01	−0.04	0.05
5. C-WSF-24 Total Score					-	0.27 **	0.32 **	0.38 **	0.23 **	0.40 **	0.39 **	0.32 **	0.42 **	−0.20 **	0.37 **	−0.03	0.31 **	−0.19 **
6. Severity Level						-	0.50 **	0.57 **	0.12 *	0.09	0.38 **	0.36 **	0.15 **	−0.13 *	0.15 **	−0.03	0.23 **	−0.06
7. Bother Level							-	0.59 **	0.08	0.18 *	0.32 **	0.31 **	0.17 **	−0.18 **	0.26 **	−0.09	0.26 **	−0.13 **
8. Functional Limitations								-	0.09	0.18 **	0.51 **	0.43 **	0.20 **	−0.12 *	0.25 **	−0.08	0.25 **	−0.12 *
9. Job Task Frequency									-	−0.46 **	0.15 **	0.19 **	0.44 **	−0.003	0.08	0.06	−0.02	0.06
10. PE of Job Tasks										-	0.37 **	0.31 **	0.45 **	−0.11 *	0.29 **	0.005	0.14 **	−0.08
11. EE Contribution to WRMSs											-	0.77 **	0.29 **	−0.12 *	0.26 **	−0.06	0.25 **	−0.09
12. EE Frequency of Encounters												-	0.21 **	−0.09	0.20 **	0.02	0.21 **	−0.12 *
13. EE Posture Frequency													-	−0.12 **	0.20 **	−0.004	0.17 **	0.02
14. Job Satisfaction														-	0.23 **	−0.18 **	0.27 **	−0.14 **
15. Job Stress															-	0.05	−0.32 **	0.13 **
16. Work Ability																-	−0.10 *	0.17 **
17. Intention to leave																	-	0.17 **
18. Work in 2 years																		-

Notes: WTP: Working through pain; SR: Social reactivity at work; DW: Demands at work; C-WSF: modified Chinese version of Workstyle short form (24 items); PE: physical exertion; EE: ergonomic exposures; * *p* < 0.05; ** *p* < 0.01.

**Table 5:** Correlations between C-WSF-24 and Job Content Questionnaire (JCQ) subscales by non-parametric Spearman’s rho.

	Skill Discretion	Decision Authority	Psychological Job Demand	Physical Job Demand	Supervisor Support	Co-Worker Support	Patient Support
1. WTP	0.04	−0.11 *	0.25 **	0.37 **	−0.13 **	−0.14 **	0.10 *
2. SR	0.10 *	−0.12 *	0.30 **	0.30 **	−0.21 **	−0.18 **	−0.13 **
3. DW	0.12 *	−0.11 *	0.42 **	0.32 **	−0.22 **	−0.23 **	−0.10 *
4. Breaks	−0.01	0.06	−0.01	0.02	0.06	0.06	0.11 *
5. Workstyle Total Score	0.11	−0.12 *	0.40 **	0.39 **	−0.21 **	−0.21 **	−0.11 *

Notes: WTP: Working through pain; SR: Social reactivity at work; DW: Demands at work; C-WSF: modified C-WSF-24. * *p* < 0.05; ** *p* < 0.01.
